# Tactile Evaluation Feedback System for Multi-Layered Structure Inspired by Human Tactile Perception Mechanism

**DOI:** 10.3390/s17112601

**Published:** 2017-11-11

**Authors:** Iza Husna Mohamad Hashim, Shogo Kumamoto, Kenjiro Takemura, Takashi Maeno, Shin Okuda, Yukio Mori

**Affiliations:** 1 Graduate School of Science and Technology, Keio University, Yokohama 223-8522, Japan; iza_husna@keio.jp (I.H.M.H.); kumamoto.no2@gmail.com (S.K.); 2 Department of Mechanical Engineering, Keio University, Yokohama 223-8522, Japan; 3 Graduate School of System Design and Management, Keio University, Yokohama 223-8526, Japan; maeno@sdm.keio.ac.jp; 4 NANJO Auto Interior Co., Ltd., Hiroshima 732-0806, Japan; shin_okuda@nanjo.co.jp (S.O.); yukio_mori@nanjo.co.jp (Y.M.)

**Keywords:** tactile sensor, tactile sensation, sensory evaluation, affective engineering

## Abstract

Tactile sensation is one type of valuable feedback in evaluating a product. Conventionally, sensory evaluation is used to get direct subjective responses from the consumers, in order to improve the product’s quality. However, this method is a time-consuming and costly process. Therefore, this paper proposes a novel tactile evaluation system that can give tactile feedback from a sensor’s output. The main concept of this system is hierarchically layering the tactile sensation, which is inspired by the flow of human perception. The tactile sensation is classified from low-order of tactile sensation (LTS) to high-order of tactile sensation (HTS), and also to preference. Here, LTS will be correlated with physical measures. Furthermore, the physical measures that are used to correlate with LTS are selected based on four main aspects of haptic information (roughness, compliance, coldness, and slipperiness), which are perceived through human tactile sensors. By using statistical analysis, the correlation between each hierarchy was obtained, and the preference was derived in terms of physical measures. A verification test was conducted by using unknown samples to determine the reliability of the system. The results showed that the system developed was capable of estimating preference with an accuracy of approximately 80%.

## 1. Introduction

A product’s tactile sensation is one of the imperative factors in making the decision to purchase a product, besides its functionality and usability [1]. Moreover, McCabe and Nowlis also prove that tactile cues significantly affect impulse purchasing [2]. Apart from that, with the presence of tactile cue, the confidence level in product evaluations increases [3]. Today product developers increasingly recognize the importance of tactile sensation as one of the added values against rivals in a competitive market [2]. For example, in development of the interiors of automobiles especially, for the parts that are directly touched by the consumer, such as seats, armrests and internal panels, the product designers choose materials from fabric to leather and plastic, and then finish them with unique kinds of texture that influence people’s subjective responses and the value of the product itself [4,5]. Generally, sensory evaluation as the mainstream method is performed to evaluate subjective responses, because this method provides direct responses from the consumer. However, this method may not be reliable for products that depend highly on time taken to the market, because the evaluation method is time consuming and also costly [6]. As the response to this issue, this paper aims to develop an assessment system that uses sensors to measure and give feedback to the developers on a consumer’s subjective responses to a product. During the purchasing process, consumers usually evaluate products through multimodal senses: sight, smell, touch, and so on, but this paper focuses on the tactile aspect of product evaluation.

During exploration of an object, humans perceive textures, hardness, shape and much more, labelled haptic information, through analyzing and integrating inputs from kinesthetic (receptors in muscle, tendon, and joint) and cutaneous (receptors in skin) systems [7,8,9,10]. These systems’ inputs are integrated and weighted in unique ways that lead to a complex human haptic perception which has a variety of factors at multiple levels of processing [8]. Previous works can be roughly classified into two categories by looking to the type of samples that are used in the product’s evaluation: (1) works that used samples that were taken from the outer layer of an object [5,11] or cut off small part from the object [12,13,14]; and (2) works that used the final end-product as the samples [15,16,17]. The latter type of sample may help us to include information from both of the two modalities (kinesthetic and cutaneous); not only the surface texture but also the tension, shape, and so on. Accordingly, this paper takes this concern into consideration by setting the object that will be evaluated as the final end-product. This is also to make sure the evaluation is not biased either to the kinesthetic or cutaneous system. Therefore, its evaluations use door armrest samples that are in their end-product form and shape, similar to the condition when they are mounted into automobiles. In addition, the multi-layered structure with different stiffness of materials inside can be taken into account.

The structure of this paper is as follows: In Section 2, the concept of the tactile evaluation feedback system will be described. This section discusses prior studies regarding the concept in developing the evaluation feedback system and the method used by this paper. Next, Section 3 explains the method of sensory evaluation for door armrest samples and the results will be discussed. Then, Section 4 describes the measurement methods of selected physical measures. Subsequently, Section 5 investigates the correlation between subjective responses and physical measures, and the evaluation feedback system will be constructed. The system will be verified by using unknown door armrest samples in Section 6, and the accuracy will be discussed. Lastly, Section 7 contains the discussion and conclusion.

## 2. Concept

In order to develop this evaluation method, human subjective responses (dependent variables) need to be collected and correlated with physical measures (independent variables) from designated sensors. This method is a basic method of quantifying or translating subjective responses to physical measures, and many previous works used this approach [5,11,13,18,19,20,21,22,23,24,25]. One of the extensions of this method is to construct a hierarchy of stages of subjective responses, for example, by classifying subjective responses into two categories, which are sensorial and affective responses [21]. All of these approaches have a similar reasoning behind them. It is based on the biological structure of human perception and cognition. When a surface is being touched, it stimulates mechanoreceptors and thermoreceptors beneath the skin [26], and the signals are mapped onto outer areas of the brain. Here, psychophysical responses of the stimuli are processed, for example, softness, roughness, warmness and so on of the object. Then, these responses trigger other areas of the brain where they are synthesized, and compared to experiences, to form affective responses [27]. In addition, Chen et al. [21] and Nagano et al. [28] suggested dividing into another two layers of affective responses which were labelled affective and preferential layers, for better understanding of complex human affective responses.

**Figure 1:**
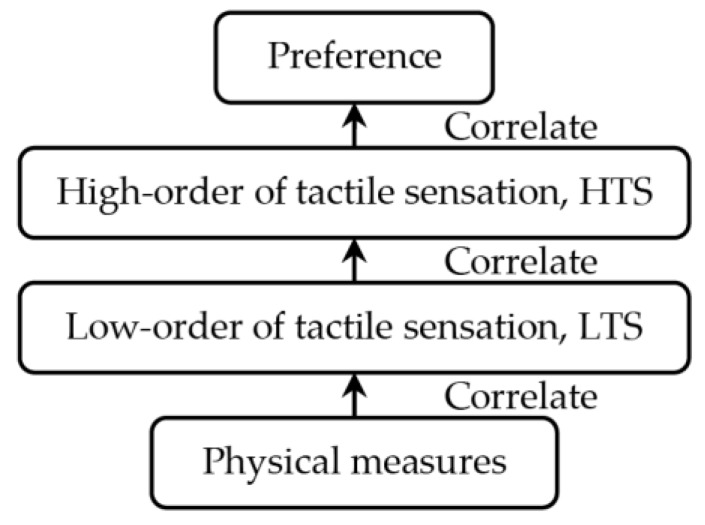
Hierarchical layered structure of tactile sensation and physical measures.

Based on the above discussion, this paper employs the concept of hierarchically layering the subjective responses and correlates the psychophysical responses with physical measures as shown in Figure 1. Note that this paper addresses subjective responses and physical measures as below. For subjective responses, psychophysical response is labelled as low-order of tactile sensation (LTS).

Affective response as high-order of tactile sensation (HTS) and preferential response as preference. LTS is a layer that includes terms which directly express physical properties in the context of tactile sense, such as *cool*, *smooth*, *soft,* and so on. On the other hand, HTS is a layer that is comprised of emotional and affective expressions, such as *fit*, *refreshing*, *luxury,* and so on. Lastly, preference is a layer that describes personal preferences which are strongly related to decision-making process while purchasing a product, such as *prefer, pleasure, comfort*, and so on.

Conventionally, haptic information can be generalized into four main aspects which are roughness, compliance, coldness, and slipperiness [21,29]. Roughness is the most studied aspect compared to others because it is the most vital aspect in differentiating between surface textures. Previous works [11,30] showed that the vibration which is evoked during dynamic interaction between skin and the object’s surface is considered to have a major role in roughness perception. Compliance has a number of ways to physically express it, and the main streams are object’s stiffness and surface deformation/Young’s modulus [29]. In a detailed investigation [31], the researchers discovered that 90 percent of the information in perceiving compliance was associated with the perception of surface deformation. Coldness is perceived due to the rate of heat loss from the skin to the object when touching it and it is mainly influenced by the material’s thermal properties, such as thermal conductivity, specific heat, and density [32]. Lastly, slipperiness is perceived through two channels: kinesthetic (when an opposite force works in the opposite direction of the motion) and cutaneous (when skin stretches tangentially to the surface) [33]. These are related to the friction that occurs during the interaction between two surfaces: skin and surface of material. In conclusion, this paper measures four physical measures which correspond to four main aspects of haptic information; vibration for roughness, bulk displacement for surface deformation for compliance, thermal properties for coldness, and friction for slipperiness. The specific measurement methods that are used in this work are reported here.

This hierarchical structure of tactile perception process mentioned above may not perfectly correspond to the process in our brain; however, it may help product development in understanding the overall structure of tactile perception mechanism in human. The correlation between LTS-HTS and HTS-Preference will be achieved from sensory evaluation data (refer Section 5). Then, the relationship between LTS and physical measures will be obtained by correlating sensory evaluation data of LTS and physical measures data. Once each relation between hierarchies is obtained, we may estimate the human preference for unknown samples by measuring the physical quantities.

## 3. Subjective Responses of Samples

### 3.1. Method

**Figure 2:**
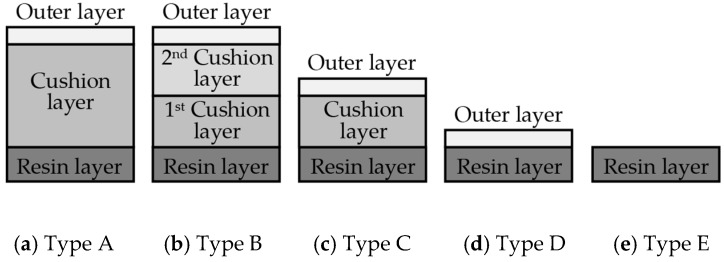
Cross-sectional structure types of door armrest samples.

A sensory evaluation with semantic differential method was carried out. 15 adults with age between their twenties and forties were asked to touch freely with their hands and evaluate 26 samples of door armrest on a seven-point unipolar scale. All participants had given their informed consent for inclusion before they participated in the study, and it was approved on 7 July 2016 by the School of Science and Technology, Keio University Research Ethics Committee (28–51). The unipolar scale is used to avoid translation problems between opposite adjectives [34]. Moreover, the scale is only defined at the endpoints to prevent varying interpretations of verbal anchors and unevenness between anchors [35]. The sensory evaluation included 22 items of adjectives listed and classified into LTS, HTS and preference as shown in Table 1. The adjectives were selected by referring to previous works [20,22,28,36,37,38] and discussion with door-armrest developers. In order to exclude visual effects from the sensory evaluation, the experiment was carried out as a blind test with samples’ details undisclosed. Table 2 shows the outer layer samples which are made of synthetic leather, genuine leather, fabric, polyvinyl chloride and resin, and the cross-sectional structure types of each sample; there are five types as shown in Figure 2.

### 3.2. Principal Component Analysis

From the survey data of sensory evaluation, principal component analysis with varimax rotation was carried out using statistical analysis software (SPSS Ver. 22, IBM, Armonk, NY, USA) to reduce the number of variables by grouping the adjectives that have strong correlation to each other into an independent semantic variable or principal component (PC). Previous research [21,39,40] has shown that the adjectives can be grouped, and principal component analysis is one of the methods used. By reducing the number of variables, they become practical and easy for interpreting the data. Table 3 and Table 4 show the results of principal component analysis for LTS and HTS, respectively. All components that have loadings higher than 0.50 are in bold (this is an arbitrary limit). 

There were six principal components for LTS extracted with 91.7% of the total variance. PC1 was interpreted as “dampness” dimension, with high loadings on *damp* and *wet*, PC2 was “coldness” dimension, with high loadings on *chilly* and *cold*. PC3 was “micro-roughness” dimension, with high loadings on *smooth* and *silky*. PC4 was “macro-roughness” dimension, with high loadings on *rough* and *bumpy*. PC5 was “hardness” dimension, with high loadings on *tough* and *hard*. Lastly, PC6 was “hollowness” dimension, with high loadings on *brittle* and *hollow*.

On the other hand, there were four principal components for HTS extracted with 92.3% of the total variance. PC1 was identified as “embracingness” dimension, with high loadings on *fit* and *embraceable*. PC2 was “refreshingness” dimension, with high loadings on *reviving* and *refreshing*. PC3 was “excitingness” dimension, with high loadings on *exciting* and *exhilarating*. Lastly, PC4 was “expensiveness” dimension, with high loadings on *cheap* (negative sign) and *luxury*. Here, *luxury* was seen to have slightly loading on PC1, in other words, *luxury* had a combination of *not cheap* and *fit.*

## 4. Data Collection of Physical Measures

As mentioned in Section 2, four physical measures (vibration, bulk displacement for surface deformation, thermal property, and friction) for each sample will be acquired in this section by a proposed tactile sensor for vibration and commercialized tactile sensors for others.

### 4.1. Vibration

During interaction between skin and object, vibration is one of the physical effects that are evoked. There are four kinds of mechanoreceptors in human glabrous skin that perceived vibration or mechanical stimuli: fast adapting, FA I (Meissner corpuscle), slow adapting, SA I (Merkel’s disc), FA II (Pacinian corpuscle) and SA II (Ruffini ending) [41]. Furthermore, each mechanoreceptor has its peculiar frequency band of vibrating stimuli, and the perceptible frequency range of human is up to 1000 Hz [42].

**Figure 3:**
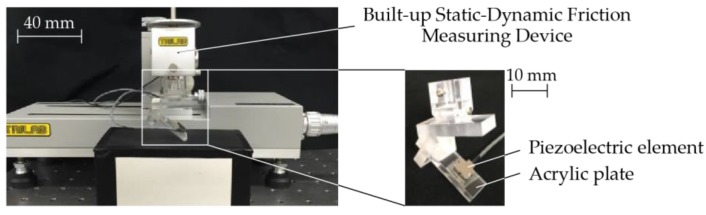
Experimental apparatus for measuring vibration.

This paper referred to previous research on the method of collecting and indexing vibrational data. Asaga et al. had collected vibrational data by tracing on the surface of samples with a piezoelectric element. Then, the vibrational data was compared with mechanoreceptors properties. As a result, two vibratory stimuli values, *I*_FA I_ and *I*_FA II_, which correspond to the firing status of FA I and FA II, were determined and used to quantify roughness [11]. Here, a 15 mm × 22 mm × 3 mm acrylic resin plate with a piezoelectric element attached to was fabricated as shown in Figure 3. Piezoelectric element is mostly used for actuating or sensing vibration in numerous research as it has simple mechanism so that it is easy to implement in any design [7,43,44,45,46]. The sensor was placed 45° to the door armrest sample and traced on with a velocity of 50 mm/s under a load of 0.49 N. Then, two values of vibratory stimuli, *I*_FA I_ and *I*_FA II_ [V^2^·Hz] which corresponded to mechanoreceptor FA I and FA II were estimated and will be used in correlating with LTS in the next section.

### 4.2. Bulk Displacement

**Figure 4:**
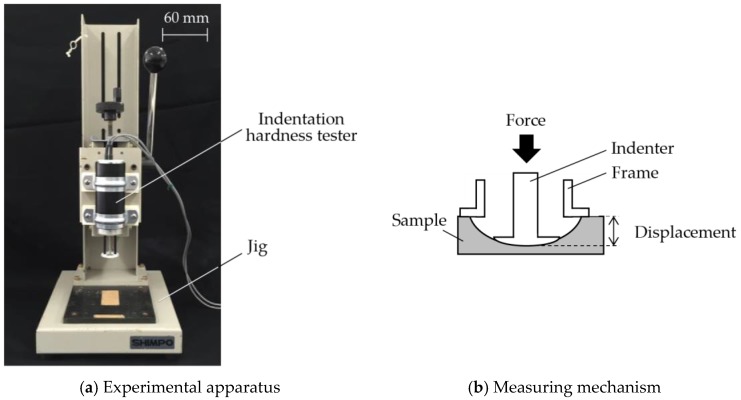
Measurement of bulk displacement.

Compliance or softness is perceived when skin is pressed on an object, and both the finger and the object deform as well as change their profile/pressure distributions [7]. However, the deformation of skin will not be measured because previous research [31] proved that 90 percent of the information in perceiving compliance is associated with the perception of surface deformation. Consequently, this paper proposes to measure bulk displacement when a fixed force is applied. By using an indentation hardness tester (TK-HS100, Tokushu-Keisoku. Co., Ltd, Yokohama, Japan) as shown in Figure 4, a sample was pressed with loads from 5 N to 30 N, with an interval of 5 N and the corresponding bulk displacements, *d* [mm] were measured.

### 4.3. Thermal Property

**Figure 5:**
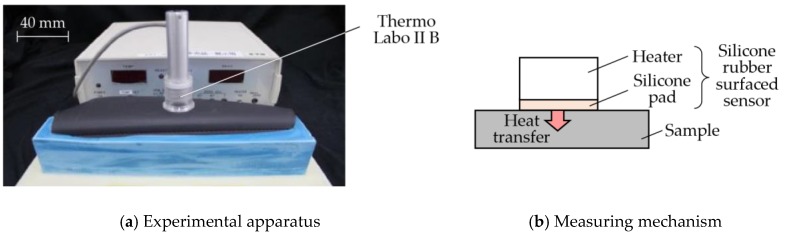
Measurement of thermal property.

Coldness or warmness is perceived when heat is transferred from or to our skin when we touch them [47]. Perception of temperature is attributed to the thermal property between skin and an object [39]. By using Thermo Labo II B (FR-07, Kato Tech. Co., Ltd., Kyoto, Japan) which is a heat flux sensor, the silicone rubber surfaced sensor with dimension of φ35 × 122 mm was preheated to 33 °C which was the average finger skin temperature, and then it was placed on the sample as shown in Figure 5. The peak heat transfer speed, *q_max_* [-] was determined. Note that the sample was left in a room with temperature of 23 °C.

### 4.4. Friction Force

**Figure 6:**
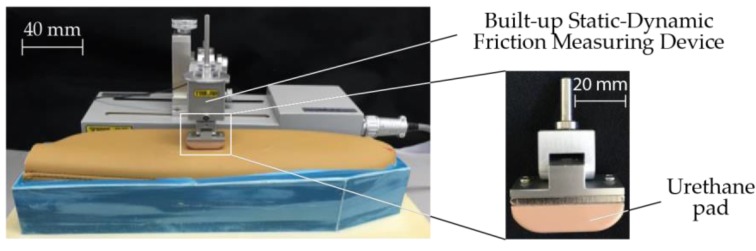
Experimental apparatus for measuring frictional force.

Slipperiness or stickiness is a perception when skin slides over on an object’s surface, and the skin stretches and adheres to the surface [39]. Furthermore, according to previous research, this perception is mainly attributed to friction forces or friction coefficients [48,49,50]. Hence, by using Built-up Static-Dynamic Friction Measuring Device (TL201Ts, Trinity-Lab. Co., Ltd., Tokyo, Japan) with a skin-like urethane pad as shown in Figure 6, frictional force was measured and its variance, *A_fric_* was computed to represent the magnitude of fluctuation of the frictional force. The sensor was placed on a sample with a preload of 1.47 N and traced with a velocity of 5 mm/s.

## 5. Correlation between Subjective Responses and Physical Measures

As mentioned in Section 2, this paper suggests hierarchically layering the subjective responses and correlating between physical measures-LTS, LTS-HTS, and HTS-Preference. For this purpose a commonly used technique, multiple linear regression analysis, is implemented [18,51,52,53,54]. This method is very simple and easy to interpret since there are multiple correlations between upper and lower layers [21,22,55].

Before conducting multiple linear regression analysis between LTS (dependent variables) and the physical measures (independent variables), principal component analysis with varimax rotation was performed to group physical measures that had strong correlation, and ensured no multicollinearity between independent variables. The result is as shown in Table 5. There were four principal components extracted with 94.9% of the total variance; PC1 was associated with bulk displacements for all load conditions, PC2 with vibratory stimuli values of *I*_FA I_ and *I*_FA II_, PC3 with peak heat transfer speed, *q_max_*, and PC4 with variance of dynamic frictional force, *A_fric_*. This result supports the concept of four main aspects of haptic information which are vibration, bulk displacement for surface deformation, thermal property, and friction.

**Figure 7:**
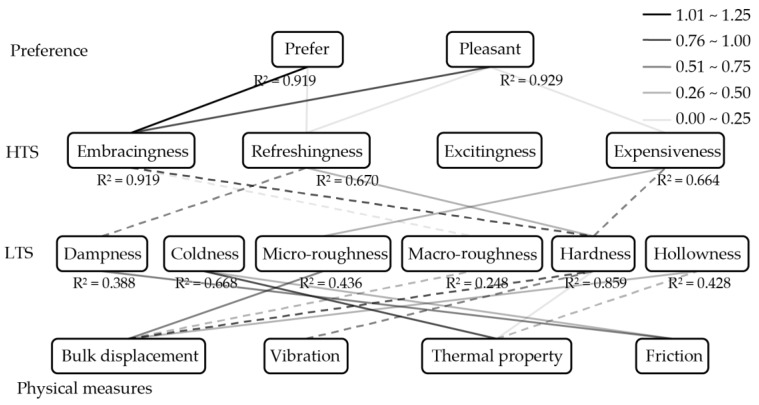
Multiple regression analysis result.

By using principal components scores, multiple linear regression with a stepwise method was conducted three times using statistical analysis software (SPSS Ver. 22, IBM, Armonk, NY, USA), and Figure 7 shows the result: (1) LTS (as dependent variables) and physical measures (as independent variables); (2) HTS (as dependent variables) and LTS (as independent variables); and (3) Preference (as dependent variables) and HTS (as independent variables). The grayscale gradient of the lines corresponds to the standardized coefficients. Moreover, the solid and dash lines indicate the positive and negative coefficients, respectively. *R^2^* is a multiple coefficient of determination that the amount of variance in the regression model, and it is a better indicator in representing strength of the relationship between independent variables and the dependent variable [56]. However, there was one component in HTS (the “excitingness” dimension) that could not successfully construct its multiple regression with components in LTS because the correlations between the “excitingness” dimension with components in LTS were not significantly strong. In order to build estimating equations for preference from physical measures, the “excitingness” dimension was not included during the construction of multiple regressions between Preference and HTS.

Furthermore, estimating equations for preference (*prefer* and *pleasure*) from principal components of physical measures were derived by substituting multiple regression equations obtained from the analysis. Hence, the feedback of subjective responses on tactile aspect for door armrest can be acquired from physical measures by using the equations as follows:

(1) $$P_{\mathrm{prefer}}\;=\;0.868\times \mathrm{Bulk}\;\mathrm{displacement}\;+\;0.380\times \mathrm{Vibration}\;-\;0.217\times \mathrm{Thermal}\;\mathrm{property}\;-\;0.0821\times \mathrm{Friction}\;+\;3.77,$$

(2) $$P_{\mathrm{pleasure}}\;=\;0.816\times \mathrm{Bulk}\;\mathrm{displacement}\;+\;0.342\times \mathrm{Vibration}\;-\;0.195\times \mathrm{Thermal}\;\mathrm{property}\;\;-\;0.0757\times \mathrm{Friction}\;+\;3.90.$$

## 6. Verification Experiment

**Figure 8:**
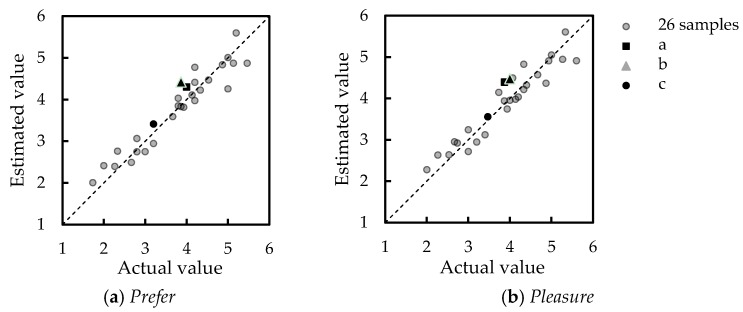
Comparison between actual and estimated scores.

In this section, the equations obtained were verified by using unknown samples of a, b, and c (refer Table 6). First, a sensory evaluation test for each sample with the same condition and participants in Section 3.1 was carried out; however, only adjectives in the preference layer were asked. Next, similar physical measures as in Section 4 for unknown samples were measured. Consequently, principal component scores for each principal component in the physical measures layer were computed by using principal component loadings. Then, the evaluation scores of *P_prefer_* and *P_pleasure_* were calculated by using estimating Equations (1) and (2), respectively. Lastly, the actual and estimated scores were compared in Figure 8. Gray plots are the 26 samples that are used in the process to derive the estimating equations, and black plots are the unknown samples. Dashed lines indicate one-to-one relationships.

The accuracy of this tactile evaluation feedback system was determined by calculating the percent error for each sample using the following equation.

(3) $$\mathrm{Percent}\;\mathrm{error}\;=\;\frac{\left|\mathrm{Estimated}\;\mathrm{score}-\mathrm{Actual}\;\mathrm{score}\right|}{\mathrm{Actual}\;\mathrm{score}}\times 100\%,$$

As a result, the maximum percent error when estimating *prefer* and *pleasure* were 20.8% and 16.2%, respectively. In other words, the developed system has the accuracy of 79% for *prefer* and 84% for *pleasure* in giving feedback on tactile evaluation.

Similarly, the percent errors when estimating *prefer* and *pleasure* for unknown samples a, b and c were computed, and the result was concluded in Table 7. Hence, by comparing the percentage error for *prefer* and *pleasure*, both have smaller percent errors compared to the developed system. Thus, this system can give estimation on unknown product’s evaluation successfully.

## 7. Discussion and Conclusions

The above physical measuring and statistical analysis results have shown that the proposed concepts of the developed assessment system can be considered adequate with slight errors. First of all, the concept of hierarchy stages of subjective responses has helped us to easily interpret the main aspect of tactile sensation that is related to the preference. From Figure 7, the “embracingness” dimension seems to have high correlation to both *prefer* and *pleasure*, compared to other HTS components. Furthermore, the “hardness” dimension shows a strong correlation to the “embracingness” dimension. Hence, the biggest influence on the preference layer can be concluded to be the “hardness” dimension in the case of tactile assessment of door armrests. However, the structure is provisional based on the kind of the object. In another study on tactile assessment of film and board materials for confectionery packaging [21], “roughness” seemed to be the most important factor to the affective layer (equivalent to HTS in this paper).

Besides, this paper suggests using the final-end product in the tactile assessment of a product that has layers of different materials. This argument is supported by the result obtained that shows the “hardness” dimension is the most important aspect in tactile assessment of door armrests. The perception of “hardness” involves both the kinesthetic and cutaneous systems [29]. This perception may not be evaluated accurately by just using only the outer layer of the sample, as the product had a layered structure.

Furthermore, this paper suggests correlating LTS with four main aspects of haptic information (roughness, compliance, coldness, and slipperiness). The physical measures for each aspects of haptic information are selected based on the kind of stimuli that evoked the receptors, and also the physical effects that occurred during the interaction between the skin and the object. Other than the physical measures that are selected in this paper (i.e. vibration for roughness, bulk displacement for surface deformation for compliance, thermal property for coldness, and friction for slipperiness), there are many other possible physical measures that are used in other studies. For example, Chen et al. chose three dimensional pictures or topography of a surface’s texture for roughness [21]. However, the result is not quite convincing. In addition, the author mentioned that there is a need for further study on the measurement of roughness and it is probably related to vibration, as mentioned in other research [30,57]. Thus, the concept proposed in this paper may help in selecting suitable physical measures.

More work is required to find the appropriate physical measures to correlate with LTS, because in this paper, several coefficient of determinations obtained from the multiple regression analysis between LTS (dependent variable) and physical measures (independent variable) are less than 0.5 (arbitrary lower limit for strong correlation), especially in the case of “micro-roughness”, “macro-roughness” and “hollowness”. Moreover, physical measure of vibration, which was expected to have correlation with roughness, was found to be not significantly correlated.

In addition, the other work is to classify people by clustering them according to their preference and then construct each group’s hierarchy structure of tactile sensation. This may help product developers in targeting their market. Before that, there is a need to increase the number of participants and vary the cohorts of people, for example, broaden the age groups.

## Figures and Tables

**Table 1:** List of adjectives.

	Adjective		Adjective
LTS	Wet	HTS	Fit
	Damp		Embraceable
	Chilly		Reviving
	Cold		Refreshing
	Smooth		Exciting
	Silky		Exhilarating
	Rough		Cheap
	Bumpy		Luxury
	Tough	Preference	
	Hard		Prefer
	Brittle		Pleasant
	Hollow		

**Table 2:** List of door armrest samples.

#1	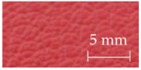	#2	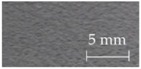	#3	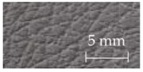	#4	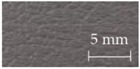
	Genuine leather		Resin		Synthetic leather		Synthetic leather
	Type A		Type E		Type A		Type B
#5	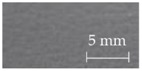	#6	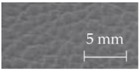	#7	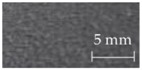	#8	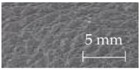
	Synthetic leather		Synthetic leather		Fabric		Resin
	Type A		Type B		Type A		Type B
#9	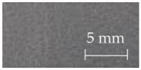	#10	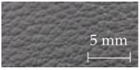	#11	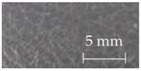	#12	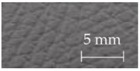
	Synthetic leather		Synthetic leather		Polyvinyl chloride		Genuine leather
	Type C		Type A		Type C		Type C
#13	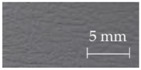	#14	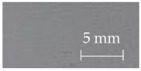	#15	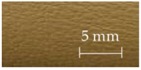	#16	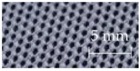
	Synthetic leather		Genuine leather		Synthetic leather		Fabric
	Type C		Type A		Type A		Type D
#17	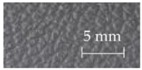	#18	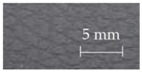	#19	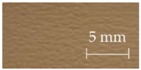	#20	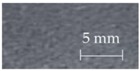
	Synthetic leather		Synthetic leather		Synthetic leather		Fabric
	Type C		Type A		Type A		Type A
#21	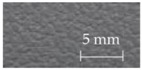	#22	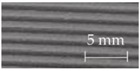	#23	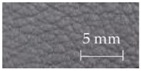	#24	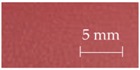
	Polyvinyl chloride		Polyvinyl chloride		Synthetic leather		Synthetic leather
	Type C		Type C		Type A		Type A
#25	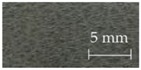	#26	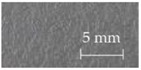				
	Polyvinyl chloride		Resin				
	Type C		Type E				

**Table 3:** Result of principal component analysis for low-order of tactile sensation (LTS).

Adjective	Principal Components					
	1	2	3	4	5	6
Wet	**0.971**	0.091	0.081	−0.014	−0.057	−0.010
Damp	**0.967**	0.098	0.125	−0.018	−0.053	−0.028
Chilly	0.088	**0.966**	0.091	0.066	0.067	0.084
Cold	0.104	**0.963**	0.091	0.066	0.112	0.038
Smooth	0.100	0.106	**0.915**	−0.176	−0.105	−0.057
Silky	0.118	0.084	**0.908**	−0.127	−0.199	0.000
Rough	−0.019	0.105	−0.093	**0.917**	0.219	0.006
Bumpy	−0.014	0.031	−0.227	**0.886**	0.216	0.100
Tough	−0.023	0.140	−0.131	0.239	**0.893**	−0.129
Hard	−0.102	0.061	−0.193	0.220	**0.887**	−0.143
Brittle	−0.101	0.069	−0.139	0.144	−0.024	**0.900**
Hollow	0.063	0.052	0.084	−0.052	−0.223	**0.892**
Eigen value	3.430	2.513	1.899	1.490	0.983	0.693
Cumulative contribution ratio	16.22	32.39	47.74	62.84	77.86	91.74

**Table 4:** Result of principal component analysis for high-order of tactile sensation (HTS).

Adjective	Principal Components			
	1	2	3	4
Fit	**0.920**	0.025	0.188	0.241
Embraceable	**0.887**	0.108	0.272	0.245
Reviving	0.075	**0.945**	0.173	0.049
Refreshing	0.057	**0.865**	0.378	0.039
Exciting	0.287	0.314	**0.858**	0.160
Exhilarating	0.256	0.322	**0.858**	0.194
Cheap	−0.216	0.018	−0.115	**−0.941**
Luxury	**0.501**	0.203	0.287	**0.695**
Eigen value	4.369	1.741	0.708	0.570
Cumulative contribution ratio	26.09	49.82	72.94	92.35

**Table 5:** Result of principal component analysis for physical measures.

Physical Measures	Principal Components			
	1	2	3	4
*d_20N_*	**0.920**	0.347	−0.124	0.083
*d_15N_*	**0.919**	0.340	−0.165	0.087
*d_25N_*	**0.910**	0.350	−0.113	0.062
*d_10N_*	**0.897**	0.308	−0.248	0.061
*d_30N_*	**0.894**	0.344	−0.106	0.032
*d_5N_*	**0.825**	0.183	−0.400	−0.021
*I*_FA I_	−0.418	**−0.840**	−0.056	0.114
*I*_FA II_	−0.477	**−0.765**	0.268	−0.103
*q_max_*	−0.250	−0.063	**0.955**	0.040
*A_fric_*	0.084	−0.021	0.035	**0.992**
Eigen value	6.931	1.061	0.951	0.545
Cumulative contribution ratio	52.77	71.77	84.54	94.87

**Table 6:** List of unknown samples.

a	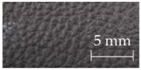	b	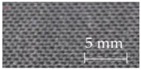	c	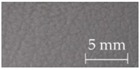
	Synthetic leather		Fabric		Genuine leather
	Type C		Type D		Type A

**Table 7:** Percent error for unknown samples.

Samples	*Prefer*	*Pleasure*
a	7.56%	13.6%
b	14.3%	12.0%
c	6.71%	2.64%
